# Impact of a behaviorally-based weight loss intervention on parameters of insulin resistance in breast cancer survivors

**DOI:** 10.1186/s12885-018-4272-2

**Published:** 2018-03-27

**Authors:** Kim L. Dittus, Jean R. Harvey, Janice Y. Bunn, Nathan D. Kokinda, Karen M. Wilson, Jeff Priest, Richard E. Pratley

**Affiliations:** 1 Department of Internal Medicine, Vermont Center on Behavior and Health, University of Vermont, Burlington, VT 05405 USA; 2 Department of Nutrition and Food Sciences, Vermont Center on Behavior and Health, University of Vermont, Burlington, VT 05405 USA; 3 Department of Mathematics and Statistics, University of Vermont, Burlington, VT 05405 USA; 4 Department of Rehabilitation and Movement Science, University of Vermont, Burlington, VT 05405 USA; 5 Vermont Cancer Center, Burlington, VT 05405 USA; 6 Florida Hospital Translational Research Institute for Metabolism and Diabetes, Sanford Burnham Prebys Medical Discovery Institute, Orlando, FL 32804 USA

**Keywords:** Breast cancer, Weight loss, Biomarker

## Abstract

**Background:**

Breast cancer survivors with excess weight are more likely to have negative breast cancer outcomes. Biomarkers related to insulin resistance may help explain this negative association. Weight loss is associated with improvements in insulin sensitivity. Our goal was to identify the impact of a behaviorally based weight loss intervention on indices of insulin resistance.

**Methods:**

Overweight, early stage breast cancer survivors who completed initial cancer therapy were enrolled in a 6 month behaviorally based weight loss intervention that included calorie reduction, exercise and behavior modification. Biomarkers related to insulin resistance were obtained at baseline and after the intervention. Results from participants who achieved ≥5% weight loss were compared to those who lost less weight.

**Results:**

Despite not having diabetes as a preexisting diagnosis prior to the study, 69% of all participants were considered to have pre-diabetes or diabetes at baseline based on American Diabetes Association definitions. Participants who achieved ≥5% weight loss had significantly lower fasting insulin, AUC insulin, and insulin resistance as measured by HOMA-IR. Beta cell function decreased as anticipated when insulin resistance improved. Additionally, leptin levels declined.

**Conclusions:**

Breast cancer survivors who achieved ≥5% weight loss demonstrated significant improvements in indices of insulin resistance. Despite an exclusion criteria of diabetes at the time of enrolment, a high proportion met criteria for pre-diabetes or diabetes at baseline. Pre-diabetes appears to be under recognized in overweight breast cancer survivors. Behaviorally based weight loss interventions can result in weight loss and improvements in biomarkers related to breast cancer outcomes and additionally may decrease the chance of developing diabetes.

**Trial registration:**

NCT01482702 4/12/2010 (retrospectively registered). https://clinicaltrials.gov/ct2/show/NCT01482702?term=Dittus&rank=4

## Background

It is estimated that 66% of breast cancer survivors are overweight or obese [1]; obese breast cancer survivors have a 30% higher risk of breast cancer and overall mortality compared to normal weight women [2, 3]. Weight gain after therapy also contributes to risk. Epidemiologic studies suggest that each 5 kg weight gain increment is associated with a 12% increase in all-cause mortality and a 13% increase in breast cancer specific mortality [4].

Insulin resistance and related pathways represent a plausible mechanistic explanation for the relationship between excess weight and negative breast cancer outcomes [5]. In pre-clinical research insulin resistance is associated with pathways implicated in cancer development and progression including the mitogen-activated protein kinase (MAPK) pathway and the phosphatidylinositol 3-kinase (PI3K) pathway [6]. Epidemiologic evidence identifies a two-fold increase in the risk of breast cancer recurrence and a three-fold increased risk of death in breast cancer survivors with the highest fasting insulin levels [5]. Adipose-derived metabolic hormones, such as the adipokines leptin and adiponectin, modulate insulin sensitivity, activate NFkB and the mTOR pathway and are also associated with breast cancer risk [7, 8].

Excess adiposity is a primary driver of insulin resistance [9], and weight loss with or without exercise can result in significant improvements in insulin resistance for post-menopausal women [10]. Positive changes in insulin-related parameters have been identified with weight loss interventions provided to breast cancer survivors. Among women who achieved ≥ 5% weight loss with a behaviorally-based weight loss intervention, a significant reduction in leptin and serum insulin were identified (*p* < 0.0001) [11]. However, measures of insulin resistance provide understanding of the mechanism of resistance beyond that provided by serum insulin alone and are likely better indicators of insulin and glucose homeostasis. In a small pilot study (*n* = 14), insulin resistance decreased as a result of a weight loss and exercise intervention provided to cancer survivors but the decline was not significant [12]. Therefore, our study aimed to evaluate the impact of a behaviorally-based weight loss intervention on insulin resistance among overweight breast cancer survivors. The secondary aim was to assess the impact of weight loss on adipokines.

## Methods

### Design and eligibility

**Fig. 1:**
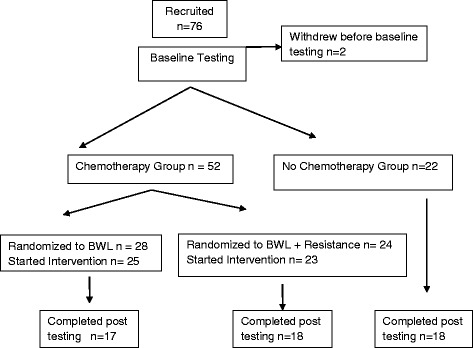
Blood was available for 70 of the 74 participants at baseline testing. Baseline and post testing blood was available for 51 of the 53 participants completing the study

A 24-week Internet-based behavioral weight loss (BWL) intervention was tested with a three arm pre-post-test study design to determine feasibility and effectiveness (Fig. 1). The primary outcome of the study was weight loss and results have been published previously [13]. Briefly, breast cancer survivors who received (CHEMO) and did not receive chemotherapy (NO CHEMO) were recruited. The women who received chemotherapy were randomized to receive the standard BWL intervention or the same intervention with added resistance training (CHEMO vs CHEMO-RT). The NO CHEMO arm served as a comparison group. The study was approved by the Institutional Review Board at the University of Vermont (UVM).

Women with early stage breast cancer completing initial oncologic intervention at least 6 weeks prior to study initiation were recruited. Oncologic intervention included surgery ± radiation and chemotherapy depending on the study arm. Eligibility criteria included a body mass index (BMI) between 26 and 50 kg/m^2^, post-menopausal status, and age ≤ 65 years. Women who had metastatic breast cancer or a preexisting diagnosis of diabetes mellitus at baseline were ineligible. For the biomarker analysis reported here, women who lost ≥5% were compared to those who lost < 5% of baseline body weight.

Power analysis was performed for the primary objective of weight loss in the original three groups. Subject numbers per group were based on the standard deviation of baseline weight (18.2 kg) and pre-post tracking correlation (*r* = 0.90) using results from a similar program delivered to women from the general population [14], with variance estimates for participants weights limited to those recruited from the local area only. Assuming a Type I error of 5% inclusion of 17 women would provide 90% power to detect a statistically significant decrease in weight of 6.95 kg overtime, which is what we found in our prior study. While 27 women /group would provide 90% power to detect a statistically significant difference in weight loss of this magnitude compared to a weight loss of 1.2 kg. Based on previous research we expect to find significant changes in biomarkers among women who lose weight. The 27 subjects/group would also provide 62% power to detect changes in Homeostatic Model Assessment (HOMA) similar to those reported by Jen et al. [15], while power is greater than 90% to detect leptin changes similar to those in this report. All sample sizes were computed using NQuery Advisor (nQuery Advisor, Verson 7.0 User’s Guide. Los Angeles CA, 2007).

### Behavioral weight loss intervention

The BWL intervention, a 24-week, behavioral, online weight control program, included calorie restriction, physical activity and behavioral modification principles [16, 17]. Participants were asked to reduce their energy intake by up to 1000 kcal/day but not to consume less than 1200 kcal/day. Individual goals were determined by multiplying baseline weight in pounds by 12 (an estimate of current calorie consumption) and subtracting 1000 cal. This reduction is known to produce a weight loss of approximately 1-2 pounds a week [18]. Moderate intensity aerobic exercise was recommended for all participants and increased gradually from 50 min/week (250 kcal) to a goal of 400 min a week (2000 kcal) [19, 20]. To achieve this goal, exercise was prescribed for ≥ 5 days/week. Brisk walking was the primary mode of activity. Individuals receiving the resistance training were additionally asked to complete 3 weekly sessions (at least 1 supervised by an exercise trainer at a YMCA) of approximately 30 min with exercises targeting both upper and lower extremities. Key behavioral strategies included stimulus control, problem solving, self-monitoring, social support and relapse prevention. Self-monitoring behavior included food and exercise journaling. An online, weekly synchronous group meeting was led by an interventionist with experience in promoting lifestyle change. The interventionist also monitored the journaling and provided individual feedback. Web-based resources to support behavior changes were available.

### Outcome assessments

Study measurements were obtained at baseline and 6 months. Weight and height were obtained on a calibrated digital scale (Scale-Tronix), and a wall-mounted Holtain stadiometer (https://holtain.co.uk/stad.php), respectively. BMI was calculated as weight(kg)/height^2^(m). Body composition, including total fat mass and fat free mass was assessed using a dual energy X-ray absorptiometry (Lunar DPX-L densitometer, Lunar Co, Madison WI). Activity-related energy expenditure (AEE) and physical activity duration was measured using BodyMedia® Body Monitoring System accelerometer worn for 7 days. AEE includes calories expended in activities of ≥ 3 metabolic equivalents (METS). Physical activity duration included the minutes of activity performed at ≥3 METS.

### Insulin resistance measures

Blood samples were collected after an overnight fast (≥ 12 h) and 24 h of inactivity at baseline and completion of the study. An Oral Glucose Tolerance Test (OGTT) was completed. Blood was drawn at 0, 30, 60, 90 and 120 min after a 75-g oral glucose load. Plasma glucose values were determined using an automated glucose oxidase method (YSI Stat Plus Analyzer). Blood was allowed to clot for 30-45 min and then centrifuged for 10 min. Serum was pipetted from the cellular layer and stored at − 80 °C.

Serum insulin was measured by using Roche Elecsys 2010 at the Laboratory for Clinical Biochemical Research, Colchester VT and is recorded in μU/ml (1 μU/ml = 6.594 pmol/L). Quality control samples measuring low, mid and high ranges were conducted. The mean intra-assay coefficient of variation for the low, mid and high range quality control samples were 2.18%, 1.64%, and 2.01%, respectively. Due to the pulsatile nature of insulin, 2 fasting measures in addition to baseline for OGTT were obtained (− 15, − 10, 0) and averaged.

Data from the OGTT were used to identify if prediabetes or diabetes was present amount participants at baseline and used the American Diabetes Association (ADA) definitions [21]. Prediabetes, or increased risk for diabetes, is defined as a fasting glucose of 100 mg/dl to 125 mg/dl (inclusive) or 2-h plasma glucose during the 75 mg OGTT of 140 mg/dl to 199 mg/dl (inclusive). Diabetes is defined as a fasting glucose of ≥ 126 mg/dl or 2-h plasma glucose during the 75 mg OGTT of ≥ 200 mg/dl.

Total glucose and insulin areas under the curve (AUC) were determined by the trapezoid method [22] using glucose and insulin measures obtained with the OGTT. The Matsuda index, a measure of insulin sensitivity, is calculated from the OGTT serum and insulin responses [23]. The Matsuda Index reflects a composite estimate of hepatic and muscle insulin sensitivity [24]. Additionally, the insulinogenic response at 30 min was calculated as an index of early insulin secretion.

The Homeostatic Model Assessment (HOMA) was used to estimate insulin resistance (IR) using fasting plasma glucose and fasting insulin values [25]. Insulin resistance is calculated with the formula: insulin (μU/ml) x glucose (mg/dl)/405. Normal IR is defined as 1. There is good correlation (0.88, 0.85 and 0.73 [26] between estimates of IR derived from HOMA and the euglyemic clamp. HOMA-%B, an estimate of beta cell function from fasting samples was also calculated. The relationship between HOMA-IR and HOMA-%B is an inverse hyperbolic curve. In the setting of normal glucose tolerance, higher degrees of insulin resistance are associated with higher beta cell function. When insulin resistance decreases, beta cell function also declines to compensate [27].

Serum leptin and total adiponectin were measured by the Laboratory for Clinical and Biochemistry Research at UVM, using a solid phase, enzyme-linked immunosorbent assay (R&D Systems). All samples from each study time point were analyzed in a single batch and run in duplicate. The mean intra-assay coefficient of variation for the low, mid and high range quality control samples were 5.27%, 5.46% and 8.75% respectively for leptin, and 5.99%, 6.28% and 7.16% respectively for adiponectin. A leptin to adiponectin ratio was calculated.

### Statistical methods

Frequencies and descriptive statistics of demographic and baseline variables were examined, including age, time from diagnosis, stage, receipt of chemotherapy, radiation, and anti-estrogen therapy, BMI at diagnosis and at study initiation, duration of physical activity at study initiation, and diagnosis of pre-diabetes and diabetes. The original three groups had similar baseline body composition. All three groups lost a significant amount of weight from baseline to post intervention measures but there were no significant differences in weight loss between groups [13]. Their exercise participation (minutes spent in moderate/vigorous physical activity) was also not significantly different. As a result, data from the three groups were combined and dichotomized by weight loss ≥ or < 5% for biomarker analysis. Five percent represents the lower threshold of loss for physical health benefits [28]. Tests of differences were conducted between the three original groups, and between those who lost ≥5% of their baseline weight versus those who did not, using Fisher’s Exact Test for categorical variables and Wilcoxon Rank Sum Test (or Kruskal-Wallis Test) for continuous variables. Analyses were conducted primarily with data from individuals who completed the intervention (*n* = 51), though comparisons of initial characteristics were made between all participants with baseline data (intent-to-treat).

A series of analysis of covariance (ANCOVA) models were tested, examining relationships between post-intervention measures and weight loss group (i.e., ≤ 5% vs. ≥ 5%) while controlling for corresponding pre-intervention measures. In the case of anthropometric measures, the only variables included in models were weight loss group and the pre-intervention measure. In the case of exercise variables (AEE and physical activity duration) and insulin parameter changes (fasting glucose, fasting insulin, AUC glucose, AUC insulin, 30-min insulin, HOMA-IR, HOMA-B, Matsuda index, leptin, and adiponectin) age, baseline BMI, weight loss group, and the corresponding pre-intervention measure were included in each model.

Finally, a two-group repeated measures analysis of variance (ANOVA) was conducted to evaluate predictors of change in insulin resistance (HOMA-IR), using weight and duration of physical activity as independent variables. Since the outcome variable was percent insulin resistance, with a distribution on one tail close to zero, a logit transformation of insulin resistance was used in the repeated measures model.

All statistical analyses were performed using SAS 9.4 (SAS Institute, Cary, NC). Across all tests, statistical significance was defined as *p* < .05 (2-tailed).

## Results

The flow of participants through the study is illustrated in Fig. 1. Seventy six participants were recruited. Baseline blood samples were available for 70 subjects. Baseline and 6-month blood samples were complete for 51 participants. Missing blood draws occurred due to difficulty obtaining access and subsequent subject refusal. Baseline medical characteristics including age, cancer characteristics and baseline BMI for those who did and did not complete the study were similar between the three groups (data not shown). As expected there were significant differences in stage and age since two groups received chemotherapy and individuals who receive chemotherapy have more advanced cancer requiring chemotherapy and are often younger.

There were no differences in age, time since diagnosis, receipt of anti-estrogen therapy or radiation between those who lost < 5% vs ≥ 5% of baseline body weight (Table 1). A greater proportion of those who lost < 5% of baseline body weight were Stage III. Those who lost < 5% also had higher BMI at time of cancer diagnosis and at study initiation and were less physically active than those who lost more weight, though these differences were not significant. The average attendance of the online synchronous group meeting for those losing < 5% baseline body weight was 14.59 ± 6.79 sessions while those who lost ≥5 attended 17.19 ± 5.31 sessions. The difference in attendance between the two groups was not significant.

**Table 1:** Baseline characteristics of participants by weight loss category

	< 5% (*n* = 20)	≥ 5% (*n* = 31)	*P*
Age, years (*M ± SD*)	54.25 ± 4.78	54.29 ± 6.55	0.757
Age range (years)	47-64	39-65	
Time from diagnosis (mos.) (*M ± SD*)	37.45 ± 19.27	31.65 ± 20.13	0.210
Range of time from Diagnosis (mos.)	10-83	9-110	
Stage			
0	2 (10%)	1 (3%)	
I	7 (35%)	21 (68%)	0.042
II	5 (25%)	7 (23%)	
III	6 (30%)	2 (6%)	
Receipt of Chemotherapy	15 (75%)	19 (61%)	0.373
Receipt of Radiation	17 (85%)	26 (84%)	1.000
Use of Endocrine Therapy	15 (75%)	27 (87%)	0.289
BMI at diagnosis (kg/m2) (*M ± SD*)	33.41 ± 7.55	31.05 ± 5.37	0.349
BMI at study initiation (kg/m2) (*M ± SD*)	34.48 ± 7.71	32.20 ± 4.63	0.531
Physical Activity Duration^a^ (min/week) at study initiation (*M ± SD*)	60.00 ± 49.72	67.10 ± 35.32	0.329
Diagnosis of pre-diabetes and diabetes based on baseline glucose values			
Pre-diabetes	17 (85%)	18 (58%)	
Diabetes	0 (0%)	1 (3%)	0.088
	85%	61%	

Categorical variables were tested using Fisher’s Exact Test. Continuous variables were tested using the Wilcoxon Rank Sum Test

^a^Physical activity performed at a moderate or greater intensity (≥ 3 METS)

### Anthropometric outcomes

The intervention resulted in significant weight loss which was the primary outcome. Using an intent to treat analysis, all participants combined lost 4.5 kg (*p* < 0.001) representing 5.2% of their baseline weight. Those completing the intervention lost an average of 5.9 kg (*p* < 0.001) or 6.9% of baseline weight. BMI, percent body fat, fat mass and fat free mass (FFM) were all significantly lower after the intervention for the entire group. Additionally, an ANCOVA using an intent to treat analysis and ANCOVA among completers revealed statistically significant decreases in weight, body mass index and fat mass post intervention for each group.

While each group lost significant weight from baseline to post study, there were no between-group differences in anthropometric parameters as a result of the intervention [13].

As expected the group who lost ≥ 5% baseline body weight had significantly greater loss of weight, BMI, % body fat and fat mass compared to those who lost < 5% (Table 2). Those who lost ≥ 5% baseline weight lost greater fat free mass than the group losing < 5% but the difference was not significant.

**Table 2:** Analysis of covariance of anthropometric measures by weight loss group

	< 5% (*n* = 20)		≥5% (*n* = 31)		*p*
	Baseline	6 Months	Baseline	6 Months	
Weight (kg) (*M ± SD*)	90.70 ± 19.84	89.74 ± 19.03	85.27 ± 13.11	76.12 ± 12.57	< 0.001
BMI (kg/m2) (*M ± SD*)	34.48 ± 7.71	34.57 ± 7.35	32.20 ± 4.63	28.78 ± 4.70	< 0.001
% Body Fat (*M ± SD*)	49.50 ± 4.48	48.73 ± 4.95	47.16 ± 4.52	42.40 ± 6.18	< 0.001
Fat Mass (kg) (*M ± SD*)	44.67 ± 13.92	43.58 ± 13.38	39.70 ± 9.63	32.18 ± 10.04	< 0.001
Fat Free Mass (kg) (*M ± SD*)	45.54 ± 7.20	45.57 ± 7.48	45.16 ± 5.22	43.64 ± 4.17	0.851

The *p*-value refers to the association of weight loss group with the 6-month measure, when the baseline measure is included in the model

Neither weight loss group experienced a significant change in AEE or moderate physical activity duration as a result of the intervention. However, those who lost ≥5% had greater active energy expenditure and spent significantly more minutes on moderate activity than those who lost < 5% (Table 3). Those who lost < 5% baseline weight exercised less after the intervention than prior though the decrease was not significant.

**Table 3:** Analysis of covariance of exercise variables, adjusted for age and baseline BMI by weight loss group

	< 5% (*n* = 20)		≥5% (*n* = 31)		*p*
	Baseline	6 Months	Baseline	6 Months	
AEE (kcal/week) (*M ± SD*)	332.37 ± 270.14	203.14 ± 161.85	364.97 ± 201.19	451.75 ± 301.73	0.012
Physical activity duration (min/week) (*M ± SD*)	60.00 ± 49.72	33.71 ± 23.30	67.10 ± 35.32	93.89 ± 69.00	0.012

The *p*-value refers to the association of weight loss group with the 6-month measure, when the baseline measure, age and baseline BMI are included in the model

### Insulin parameter outcomes

At baseline many participants were identified to have impaired glucose, either prediabetes or diabetes, based on fasting glucose or glucose at 120 min after the OGTT (Table 1). A larger proportion of those losing < 5% baseline body weight were considered to have prediabetes or diabetes (85%), than those losing ≥ 5% baseline weight (62%), however the differences were not significant. No participant carried a diagnosis of diabetes at the time of study entry and no one was receiving medication for diabetes.

There were no between-group differences at baseline for any biomarker. No biomarker measures changed significantly from baseline for participants losing < 5% of baseline weight. Those who lost ≥5% had significantly lower fasting serum insulin, AUC insulin, 30 min insulin secretion, HOMA-IR, and HOMA-B than those losing < 5% body weight (Table 4). There were no significant differences in fasting glucose or AUC glucose between the two groups. The Matsuda index, a measure of insulin sensitivity, was significantly higher after the intervention than baseline in the group losing ≥5% baseline weight compared to those who lost < 5% .

**Table 4:** Analysis of covariance of insulin parameter changes, adjusted by age and baseline BMI, by weight loss group

	< 5% (*n* = 20)		≥5% (*n* = 31)		*p*
	Baseline	6 Months	Baseline	6 Months	
Fasting glucose (*M ± SD*)	94.05 ± 6.73	93.72 ± 7.89	93.40 ± 10.33	96.22 ± 14.46	0.328
Fasting insulin (*M ± SD*)	8.54 ± 4.12	10.72 ± 6.73	9.88 ± 7.08	6.96 ± 4.40	0.005
AUC glucose (*M ± SD*)	7956.75 ± 3140.77	8079.25 ± 3267.80	6883.71 ± 3389.54	6542.26 ± 3720.90	0.261
AUC insulin (*M ± SD*)	6756.94 ± 2422.85	7248.23 ± 2176.00	8009.73 ± 4362.87	6586.39 ± 4579.06	0.026
30-min insulin (*M ± SD*)	45.87 ± 23.37	55.63 ± 33.42	58.38 ± 34.42	48.08 ± 35.09	0.039
HOMA-IR (%) (*M ± SD*)	1.17 ± 0.49	1.39 ± 0.84	1.36 ± 0.91	0.97 ± 0.58	< 0.001
HOMA-B (%) (*M ± SD*)	95.28 ± 27.88	106.74 ± 46.13	105.94 ± 41.44	85.17 ± 33.57	0.011
Matsuda index (insulin sensitivity) (*M ± SD*)	4.14 ± 1.30	3.64 ± 1.20	4.70 ± 2.79	5.87 ± 2.92	0.001
Leptin (ng/mL) (*M ± SD*)	50.07 ± 24.02	46.31 ± 23.54	40.17 ± 16.47	23.09 ± 15.23	< 0.001
Adiponectin (mcg/mL) (*M ± SD*)	10.86 ± 40.65	10.82 ± 45.13	13.18 ± 5.36	13.19 ± 5.07	0.319
Leptin/Adiponectin (*M ± SD*)	4.92 ± 2.18	4.53 ± 1.76	3.94 ± 2.94	2.17 ± 1.89	< 0.001

The *p*-value refers to the association of weight loss group with the 6-month measure, when the baseline measure, age and baseline BMI are included in the model. HOMA-IR (%) was logit-transformed prior to analysis of covariance. Analysis of covariance of the Matsuda index was

HOMA-IR – estimate of insulin resistance

HOMA-B – estimate of beta cell function

Post intervention leptin levels were significantly lower in those losing ≥5% than those who lost < 5%. There were no differences in adiponectin between groups. However, the leptin/adiponectin ratio was significantly different between groups.

The repeated measures ANOVA assessing change in insulin resistance as measured by HOMA-IR indicated a significant association between weight change and the dependent variable [*F*(1,34) = 47.98, *p* < 0.001], but not between duration of physical activity and change in insulin resistance [*F*(1,34) = 0.46, *p* = 0.501].

## Discussion

Breast cancer survivors with excess weight have negative outcomes compared to those with normal weight [2, 4]. Cell signaling changes associated with insulin resistance and related parameters offer a plausible mechanism for the negative outcomes. It is estimated that lowering serum insulin by 25% may improve survival by 5%, the same order of magnitude as adjuvant chemotherapy [29]. Weight loss improves parameters of insulin resistance in the general population [10]. Likewise the current study identifies favorable shifts in insulin parameters among breast cancer survivors who achieve weight loss.

Despite excluding individuals with a preexisting diagnosis of diabetes and use of diabetes medications, a high proportion of participants had prediabetes and diabetes at baseline based on ADA definitions [21]. The almost 70% incidence of prediabetes and diabetes at baseline among breast cancer survivors was higher than expected. In a general population of postmenopausal women receiving a similar BWL intervention, 33% had impaired fasting glucose at baseline [10]. Despite similar BMI at baseline, incidence of impaired glucose metabolism was higher in participants losing < 5% weight, though not significantly different from those who lost ≥5% weight. Prediabetes or diabetes may have made weight loss more difficult for those losing less weight [30].

Insulin and related factors are mitogenic and can stimulate cell signaling in an aberrant manner thus enhancing growth factor-dependent cell proliferation [6, 31]. The weight loss intervention resulted in significant improvements in both fasting insulin and insulin response to a glucose challenge (AUC insulin). Fasting insulin declined by 29%, exceeding that suggested to improve survival [29]. Significant improvements in serum insulin in breast cancer survivors achieving ≥ 5% weight loss have been identified previously where participants reaching this degree of weight loss experienced 21.9% decrease in serum insulin with weight loss and exercise [11]. Greater weight loss results in even further improvements in fasting serum insulin. A weight loss intervention which included prepackaged entrees resulted in 14% weight loss and a 42.9% decrease in insulin [32]. Furthermore, we identified significant diminution of insulin response to a glucose challenge as AUC insulin after an OGTT was significantly lower in those losing more weight. Significant improvement in both parameters suggests that breast cancer survivors with greater weight loss are exposed to less circulating insulin.

Insulin resistance is an independent predictor of chronic disease including cancer [33, 34]. Insulin resistance is associated with increased insulin-like growth factor I (IGF-1). Insulin and IGF-1 can both result in decreased Sex Hormone Binding Globulin and therefore higher levels of bioavailable estradiol [31]. Insulin resistance is also associated with increased reactive oxygen species [31]. In our study, insulin resistance as measured by HOMA declined in those who achieved ≥ 5% weight loss but not in those who did not lose weight. As expected, HOMA B, a measure of beta cell function also declines with weight loss since beta cell function decreases to compensate for declines in insulin resistance. The Matsuda index which assess hepatic and muscle insulin sensitivity improved with weight loss in the current study.

Other weight loss interventions with breast cancer survivors reported varied success with modifying insulin resistance [11, 15]. The current study resulted in a 29% improvement in insulin resistance as measured by HOMA. Insulin resistance was assessed in breast cancer survivors randomized to differing weight loss interventions [15], but despite achieving 9.5% weight loss among those attending individual counseling and group meetings, insulin resistance did not improve. In a population of breast cancer survivors with a BMI similar to the current study, Campbell et al. identified a non-significant 30% improvement in HOMA-IR after a 24-week BWL intervention [12]. The lack of significance in both studies is likely due to the limited sample size. In a larger study, Thomson et al. identified a significant decrease in insulin resistance measured by HOMA with both low carbohydrate and low fat diets provided to breast cancer survivors [35]. The improvement in insulin resistance in this study and other weight loss interventions for breast cancer survivors exceeds the 17% improvement identified with the use of metformin [36]. Neither fasting glucose nor AUC glucose changed as a result of the intervention. This is not surprising given that glucose levels are tightly controlled by insulin.

Adipocytes secrete leptin and other adipokines [37]. Leptin has been linked to breast cancer outcomes [38, 39]. Leptin is independently associated with insulin resistance [40] and is angiogenic [38], which may help explain why breast cancer survivors with excess weight have negative breast cancer outcomes. We identified significant decreases in leptin after weight loss, similar to other studies [11, 15, 32, 41, 42]. Leptin levels in normal weight individuals range from 5 to 10 ng/ml [43]. Baseline leptin levels in the overweight population of the current study were 4 times higher and decreased by over 40% with weight loss. The decrease in leptin in this and other weight loss interventions for breast cancer survivors exceeds the improvements identified with the use of Metformin [36]. The study population was likely too small to see changes in adiponectin. Other weight loss interventions for breast cancer survivors have also failed to measure significant changes in adiponectin [42].

Exercise and weight loss are both associated with decreased insulin resistance and may have contributed to the improvements in insulin parameters in those losing ≥5% of baseline body weight as this group participated in significantly longer duration of moderate activity than those who lost < 5% weight. However, multiple regression analysis identified that weight loss alone significantly explained improvements in insulin resistance. Other studies have shown improvements in insulin parameters with exercise alone [44, 45]. However, among postmenopausal women without a history of breast cancer, exercise added to weight loss did not further improve fasting insulin or insulin resistance [10] and is consistent with our findings.

The study has several limitations. The attrition rate made comparisons between the planned treatment groups difficult. Statistical analysis was performed on data from only those who completed the study rather than using intention to treat analysis since our goal was to compare differences in insulin parameters between those who were and were not successful with weight loss. It is possible that those who discontinued the study have different insulin resistance parameters at baseline and response to weight loss than those who completed the intervention. The study population is primarily Caucasian and results may differ by racial or ethnic group. A subset of participants received resistance training, which is known to improve insulin resistance [46, 47], however, they did not experience increased strength or muscle mass from baseline. Therefore, it is unlikely that strength training contributed to decreased insulin resistance, an assumption borne out in that exercise duration was not related to change in insulin resistance. The accelerometer underestimates physical activity such as cycling or swimming, however walking was the exercise encouraged by the intervention. The study population included a large proportion of individuals with pre-diabetes or diabetes despite exclusion criteria for preexisting diabetes.

## Conclusion

Insulin resistance is a risk factor for increased risk of recurrence and poor prognosis in breast cancer survivors. However, individuals who achieve a minimum of 5% weight loss had significantly lower insulin resistance and decreased fasting insulin levels. If weight loss can be achieved, breast cancer survivors have the potential to improve insulin-related parameters, which may decrease chances of negative breast cancer outcomes. Additionally, overweight breast cancer survivors may have a higher than expected incidence of unidentified diabetes and metabolic changes suggestive of pre diabetes, and achieving weight loss may decrease their risk of diabetes. Increased screening for diabetes may be warranted in the overweight or obese breast cancer survivor population. Research is needed to identify if changes in insulin pathways or other biomarkers translate into improved survival and to identify effective weight loss interventions for breast cancer survivors who have difficulty losing weight.

## Abbreviations

ADA: American Diabetes Association
AEE: Activity-related Energy Expenditure
ANCOVA: Analysis of Covariance
ANOVA: Analysis of Variance
AUC: Area Under the Curve
BMI: Body Mass Index
BWL: Behavioral Weight Loss
HOMA: Homeostatic Model Assessment
IR: Insulin Resistance
METS: Metabolic Equivalents
OGTT: Oral Glucose Tolerance Test
UVM: University of Vermont
